# Gas-Forming Psoas Abscess Secondary to Lumbar Spondylodiscitis

**DOI:** 10.7759/cureus.14388

**Published:** 2021-04-09

**Authors:** Yi Xiang Tan, Wan Lye Cheong, Teck Siong Fong

**Affiliations:** 1 Orthopaedic Surgery, Putrajaya Hospital, Putrajaya, MYS

**Keywords:** spondylodiscitis, psoas abcess, back ache

## Abstract

Lower back pain is a common presentation in clinical practice. Although most are musculoskeletal in nature, occult spine infection such as spondylodiscitis is commonly missed due to its insidious onset and non-specific symptoms. We report a case of a 63-year-old diabetic woman who presented to our institution’s emergency department with altered mental status, nausea, and vomiting. She had a fall one month prior with persistent lower back-pain of increasing intensity. Initial laboratory data revealed an elevated leukocyte count with neutrophil predominance. Plain radiographs showed extensive gas shadows over the chest wall, abdomen, left thigh, and left knee. CT scan revealed L2 compression fracture with spondylodiscitis at L1/L2, left psoas abscess, and extensive subcutaneous emphysema. Open abscess drainage with extensive wound debridement was performed. Intra-operative pus, as well as blood cultures, yielded Escherichia coli. Unfortunately, the patient succumbed to the infection on the seventh day of admission secondary to multi-organ failure.

## Introduction

Septic discitis is a primary infection of the disc space caused by pyogenic organisms [1]. Historically tuberculosis used to represent the major cause of spine infection. In the 1950s, studies showed about 59% of the cases were caused by Mycobacterium tuberculosis [2]. However, the recent trend shows a shift towards pyogenic infection with Staphylococcus aureus becoming the most frequent organism causing vertebral infections, accounting for 20% to 84% of all cases [3]. Additionally, Enterobacteriae spp. are found in 7% to 33% of pyogenic vertebral infections of which Escherichia coli is the most common pathogen [3]. Complications with high mortality rate can occur if the diagnosis is delayed. Despite the severity of the infection, there is a lack of concrete evidence which can provide a guideline for the treatment of this infection. A systematic review of 20 articles showed very low evidence in producing a definitive guideline [4]. Hence, we present a case of a gas-forming psoas abscess which developed from a primary infection at the lumbar intervertebral disc. The aim of this case report is to highlight the importance of a systematic approach to a patient who presents with infective signs with a history of back or hip pain to prevent under-diagnosis of spondylodiscitis. Morbidity and mortality arising from spondylodiscitis can be reduced with early diagnosis and prompt treatment.

## Case presentation

A 63-year-old female with underlying diabetes mellitus presented to our institution’s emergency department with altered mental status, nausea, and vomiting. One month prior to presentation she had a fall and has been suffering from persistent lower back pain, progressively increasing in severity. Three days before presenting to our hospital, she was unable to ambulate due to severe backache and new onset left hip pain. Subsequently, she developed chills, nausea, vomiting, diarrhea, and altered mental status. On presentation to our institute, she had a temperature of 36.3 degree Celsius, pulse rate of 121 beats/min, blood pressure of 119/74 mmHg, and a respiratory rate of 18 per minute. Physical examination revealed an increasingly distressed female with severe pain over the left flank and left hip. There was also extensive subcutaneous emphysema over the bilateral chest wall and left flank, extending distally up to her left calf. Initial laboratory data revealed an elevated leukocyte count of 30.0 X 10^9/L with 96% neutrophils. Plain radiographs showed extensive gas shadows over the chest wall, abdomen, left thigh, and left knee.

**Figure 1:**
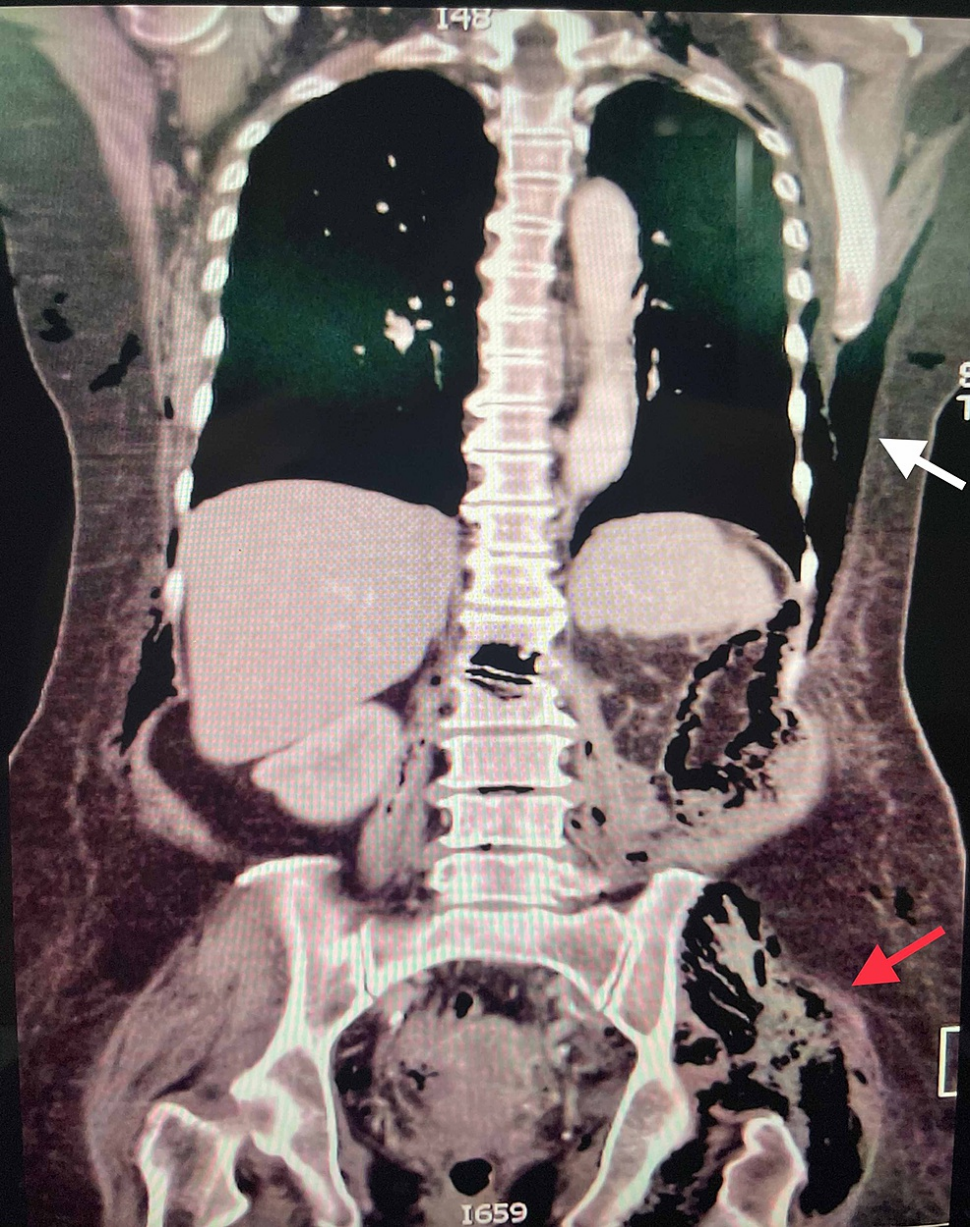
Computed tomography scan showing an abscess of the left psoas muscle (red arrow) with extensive subcutaneous emphysema (white arrow).

**Figure 2:**
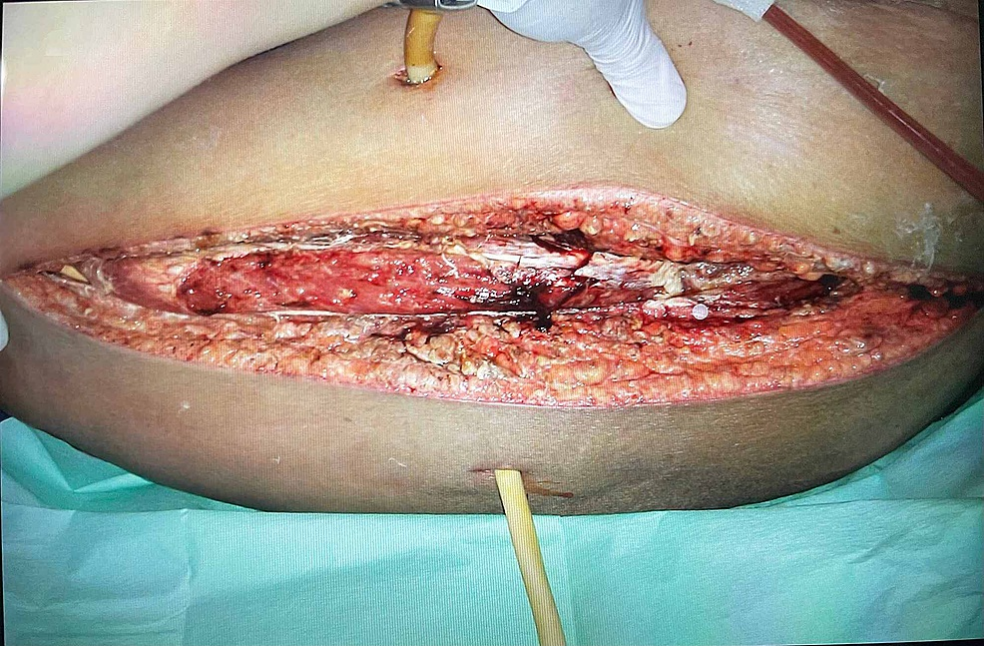
Left thigh wound after debridement.

A non-contrasted computed tomography of the thorax/abdomen/pelvis was performed to further delineate the source of infection. The CT scan revealed an L2 compression fracture with spondylodiscitis at L1/L2, left psoas abscess, and extensive subcutaneous emphysema involving the thorax, abdomen, and all compartments of the left lower limb. There was destruction of the inferior half of the L1 vertebral body with areas of necrosis within the L1 vertebral body. Pockets of air were seen in the L1/L2, L3/L4, and L4/L5 intervertebral spaces. Air was also noted within the spinal epidural space from the level of T12-S1 vertebral bodies. Other incidental findings include a fatty liver and a left lower pole renal cortical cyst (Figure 1). Emergency open abscess drainage with extensive wound debridement was performed (Figure 2). Large collections of pus were noted over the retroperitoneal region, extending to both the anterior and posterior compartment of the left thigh. A gram stain from the drainage revealed Gram-negative bacilli. Cultures of the intra-operative pus as well as blood cultures both yielded Escherichia coli. The constellation of findings concurs with gram-negative sepsis. She underwent another wound debridement five days later. Her clinical condition deteriorated despite antibiotic therapy based on sensitivity test with clindamycin, imipenem, and vancomycin. Ultimately she passed away on the seventh day of admission secondary to multi-organ failure.

## Discussion

Septic discitis is defined as a primary infection of the disc space caused by pyogenic organisms [1]. It is difficult to diagnose and often delayed due to lack of localizing signs and symptoms. The presentation may be acute or insidious, and presence of radicular or spinal compressive symptoms should raise one’s suspicion. These infections are initially confined to the intervertebral disc and according to reported literature, the lumbar spine is the most frequent location of septic discitis [5]. Although most reports of spontaneous discitis are predominantly in children, this condition is also encountered with some frequency in adults. Hematogenous spread of an infective organism is the most likely method of inoculation of the disc in spontaneous discitis [6]. The avascularity of the nucleus pulposus prevents the colonization of organisms in this region, however, once seeding had occurred, this disc space is particularly vulnerable to infection [7]. A study in 2002 revealed that in 62% of patients, the presence of concurrent infection elsewhere may pose a risk factor for disc space infection, further supporting the hematogenous mode of spread of this infection [5]. Although reports state that Staphylococcus aureus is the most commonly isolated organism, discitis secondary to Escherichia coli infection is not uncommon, as seen in this case [1,8]. This organism is often associated with urinary or gastrointestinal tract infections, advanced age, immunosuppression, and diabetes [3]. The earliest detectable radiological sign of a disc space infection is narrowing of the intervertebral disc space; however, this is rarely seen in isolation. Most patients would already have advanced radiological changes during presentation such as eburnation of subchondral bone of the adjacent vertebral bodies and irregularity of adjacent vertebrae visible on CT scans [8]. 

For our patient, it is possible that the psoas abscess developed as a sequela of septic discitis. Spinal sources are the third most common cause of secondary psoas abscess after gastrointestinal tract and urogenital infections [9]. Common pathogens encountered are mixed, predominated by Escherichia coli, Bacteroides spp., Staphylococcus, and Streptococcus [10]. Psoas abscess remains a rare clinical entity due to the non-specific nature of the presentation and subsequent delayed diagnosis. However, this is an important and potentially life-threatening condition. A high index of suspicion is required in the clinical diagnosis of this condition [11]. Supportive laboratory findings in psoas abscess include leukocytosis, an elevated erythrocyte sedimentation rate (ESR), elevated blood urea nitrogen levels, and pyuria [9]. The extent of infection usually corresponds with the rise in C-reactive protein (CRP). CT scan was performed in this case and this imaging modality has been established as the gold standard for diagnostic imaging of psoas abscess [12]. Standard X-rays and ultrasound have not been proven to be of adequate sensitivity, compared to the diagnostic rate of 88%-100% reported with CT scans [12]. MRI plays a limited role in the diagnosis of psoas abscess due to the associated higher cost and greater patient discomfort as well as the possibility of missing gas inclusions [13]. A known but rare soft tissue infection in the retroperitoneal abscess is the formation of crepitant myositis. These are categorized as clostridial and non clostridial-related myositis, usually a mixed aerobic and anaerobic infection in the latter [14]. In this patient, the presence of crepitant myositis of her left lower limb is a rare extension of a retroperitoneal abscess. The possible anatomical course of this extension may follow the pathway from the greater or lesser sciatic foramen into the hip and buttocks, or from the obturator or femoral canal into the thigh and hip, extending distally [15]. Prompt treatment is mandatory following the diagnosis of psoas abscess. Drainage of abscess and administration of intravenous antibiotics are essential for the treatment of retroperitoneal abscess [16]. In view of the secondary nature of the psoas abscess in this patient, it is essential to combine abscess drainage with treatment of the primary focus of infection [17]. Infection processes involving the spine may lead to spinal instability due to the destruction of the vertebral body and intervertebral space. Early therapeutic intervention corresponds to good prognosis in psoas abscess. Antibiotic therapy for pyogenic discitis usually takes a course of six weeks, with the recommended intravenous antibiotic for two to four weeks with a subsequent oral regimen, provided pus has been drained and CRP is reducing in trend [18-20]. At present, the mortality rate of secondary psoas abscess stands at 18.9% [10], and these have been associated with delay or inadequate diagnosis and treatment. The importance of a systematic approach to a patient who presents with signs of infection with a history of back or hip pain cannot be overemphasized. Although most cases of lower back pain are attributed to mechanical causes, a thorough history and physical examination may reveal a more sinister cause, such as spine infection. In this case, the delay in the diagnosis and treatment of the primary infection may have led to the dissemination of an initially contained infection, making it refractory to treatment even after drainage and intravenous antibiotics.

## Conclusions

Lower back pain is a common presentation in orthopaedic practice, and should not be treated lightly. A systematic examination and routine laboratory, as well as radiological investigations, should be performed in patients with a high index of suspicion. A delay in diagnosis and treatment may lead to severe morbidity or even mortality in patients with spondylodiscitis.
